# Overexpression of tripartite motif-containing 47 (TRIM47) confers sensitivity to PARP inhibition via ubiquitylation of BRCA1 in triple negative breast cancer cells

**DOI:** 10.1038/s41389-023-00453-7

**Published:** 2023-03-11

**Authors:** Fengen Liu, Binhui Xie, Rong Ye, Yuankang Xie, Baiyin Zhong, Jinrong Zhu, Yao Tang, Zelong Lin, Huiru Tang, Ziqing Wu, Heping Li

**Affiliations:** 1 Department of General Surgery III, the First Affiliated Hospital of Gannan Medical University, Ganzhou, 341000 P. R. China; 2 Department of General Surgery I, the First Affiliated Hospital of Gannan Medical University, Ganzhou, 341000 P. R. China; 3 Ganzhou Key Laboratory of Hepatocellular carcinoma, the First Affiliated Hospital of Gannan Medical University, Ganzhou, 341000 China; 4 Guangdong Province Key Laboratory for Biotechnology Drug Candidates, School of Life Sciences and Biopharmaceutics, Guangdong Pharmaceutical University, Guangzhou, China; 5 Department of Pathology, Cancer Center, Integrated Hospital of Traditional Chinese Medicine, Southern Medical University, Guangzhou, Guangdong 510310 China; 6 Cheerland Watson Precision Medicine Co. Ltd, Shenzhen, 518036 P. R. China; 7 Guangdong Provincial Key Laboratory of Molecular Tumor Pathology, Guangzhou, 510515 China; 8 Department of Pathology, School of Basic Medical Science, Southern Medical University, Guangzhou, 510515 China; 9 Department of Medical Oncology, the First Affiliated Hospital of Sun Yat-sen University, Guangzhou, 510080 P. R. China

**Keywords:** Breast cancer, Tumour biomarkers

## Abstract

Triple-negative breast cancers (TNBC) frequently harbor defects in DNA double-strand break repair through homologous recombination (HR), such as BRCA1 dysfunction. However, less than 15% of TNBC patients were found to carry BRCA1 mutation, indicating that there are other mechanisms regulating BRCA1-deficient in TNBC. In the current study, we shown that overexpression of TRIM47 correlates with progression and poor prognosis in triple-negative breast cancer. Moreover, we demonstrated that TRIM47 directly interacts with BRCA1 and induces ubiquitin-ligase-mediated proteasome turnover of BRCA1, subsequently leads to a decrease of BRCA1 protein levels in TNBC. Moreover, the downstream gene expression of BRCA1, such as p53, p27, p21 was significantly reduced in the overexpression of TRIM47 cell lines but increased in TRIM47-deleted cells. Functionally, we found that overexpression of TRIM47 in TNBC cells confers an exquisite sensitivity to olaparib, an inhibitor of poly-(ADP-ribose)-polymerase (PARP), but TRIM47 inhibition significantly confers TNBC cells resistance to olaparib both in vitro and in vivo. Furthermore, we showed that overexpression of BRCA1 significant increase the olaparib resistance in TRIM47-overexpression-induced PARP inhibitions sensitivity. Taken together, our results uncover a novel mechanism for BRCA1-deficient in TNBC and targeting TRIM47/BRCA1 axis may be a promising prognostic factor and a valuable therapeutic target for TNBC.

## Introduction

Breast cancer is the most common cancer among women. There are about 2.1 million newly diagnosed female breast cancer cases in 2018, which accounting for almost 1 in 4 cancer cases among women worldwide [1]. Until now, the main curative treatment for breast cancer patients is to adopt precise and comprehensive treatment principles, combined with various treatment methods, including surgery and chemotherapy, so as to effectively improve the curative effect and improve the quality of life of patients [2–4]. However, the therapeutic effect and prognosis for breast cancer patients is still far from optimistic.

Incidence and mortality rates of breast cancer are expected to increase significantly due to its tumor heterogeneity and genetic factors, including type LminalA, LminalB, three negative breast cancer (TNBC), and Her2 positive breast cancer [5–7]. Among which, TNBC, account for about 15% of all breast cancer patients, is a malignant tumor that characterized by pathological examination showing that estrogen receptor (ER), progesterone receptor (progesterone receptor, PR) and human epidermal growth factor receptor 2 (human 2) are negative, and is considered to be an independent clinical pathological type [8, 9]. Compared with other types of breast cancer, TNBC is characterized by higher invasiveness, worse prognosis, earlier recurrence, and distant metastasis [10]. There have been significant improvements in the outcome of other subtypes of breast cancer, including ER-positive/HER2 overexpressed tumors, attributed to the addition of targeted therapy, including hormonal agents and trastuzumab [11, 12]. However, no specific targeted agents are currently available for the treatment of TNBC [13, 14]. Therefore, identifying effective treatment strategies and understanding the molecular mechanisms responsible for the pathogenesis of TNBC are needed urgently.

The human breast cancer susceptibility gene 1 (Breast Cancer susceptibility gene 1, BRCA1), which located in human chromosome 17q21, encoding a protein consisting of 1863 amino acids [15]. BRCA1 contains two functional domains: the amino terminal has a ring domain of ubiquitin ligase activity, which mediates the interaction between protein and DNA, protein and protein; and the carboxyl terminal has two repetitive BRCT domains, which can bind to DNA response factors, participate in DNA damage repair and regulate checkpoints in the cell cycle [16]. Mutation of BRCA1 cause the protein level reduced and promotes chromosome instability, subsequently leads to cell carcinogenesis [17]. It has been reported that mutation of BRCA1 is closely related to the occurrence poor prognosis of breast cancer. Lee EY found that BRCA1 mutation carriers was mostly diagnosed as TNBC and the risk and survival of breast cancer patients carrying BRCA1 mutations is worse than that of other subtypes of breast cancer [18]. Byrski et al. found that TNBC and breast cancer patients which containing BRCA1 mutation shown the same characteristics that both sensitive to DNA damage drugs such as platinum [19]. Furthermore, PARP inhibitors, which have been shown to play a major role in the early clinical trials of BRCA-deficient breast cancer, can enhance the efficacy of TNBC in combination with chemotherapeutic agents [20]. Therefore, it is suggesting that TNBC is closely related to BRCA1 mutation.

It has been reported that less than 15% of TNBC patients were found to carry BRCA1 mutation, however, deregulation of BRCA1 was found in multiple TNBC patients, indicating that there are other mechanisms regulating BRCA1-deficient in TNBC. Tripartite motif family proteins 47 (TRIM47), a member of E3 ubiquitin ligase containing zinc finger domain, b-box, and coiled-coil domain, is located in the 17q24-25 region where amplification often occurs in many tumors [21]. It has been reported that TRIM47 is upregulated in astrocytoma [21], prostate cancer [22], and non-small cell lung cancer [23], and overexpression of TRIM47 promotes the malignant development of tumors. However, whether TRIM47 is involved in the ubiquitination modification of BRCA1 and reducing the level of BRCA1 protein in TNBC remains unclear.

## Results

### TRIM47 overexpression correlates with progression and poor prognosis in triple-negative breast cancer

**Fig. 1:**
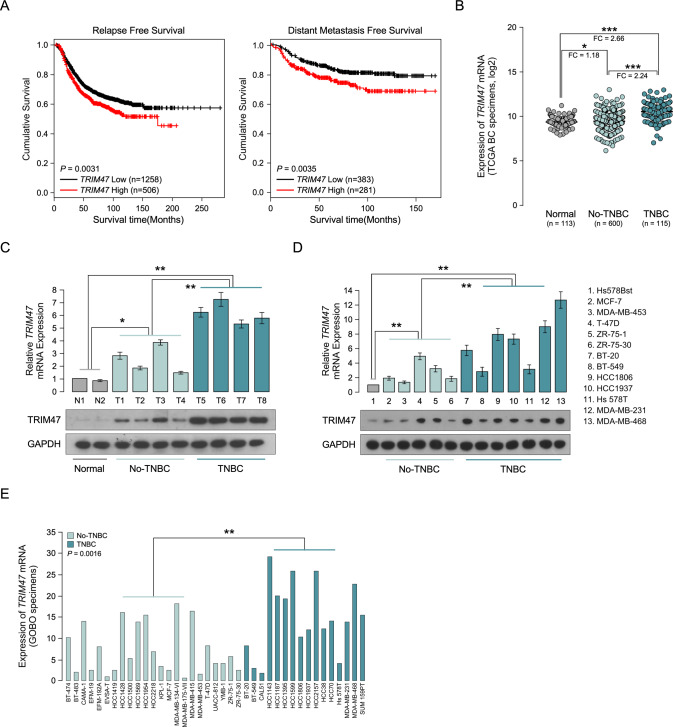
TRIM47 overexpression correlates with progression and poor prognosis in triple-negative breast cancer. **A** Kaplan–Meier analysis of relapse-free survival and distant metastasis free survival from a public dataset for breast cancer patients with low TRIM47 expression or high TRIM47 expression. **B** mRNA analysis of TRIM47 in TCGA public dataset for normal, no-triple-negative breast cancer and triple-negative breast cancer patients. **C** Real-time PCR(up) and WB(down) analysis of TRIM47 expression in 2 freshly collected normal, 4 no-triple-negative breast cancer and 4 triple-negative breast cancer tissues. **D** Real-time PCR(up) and WB(down) analysis of TRIM47 expression in 6 no-triple-negative breast cancer cells and 6 triple-negative breast cancer cells. **E** mRNA analysis of TRIM47 in GOBO public dataset for normal, no-triple-negative breast cancer and triple-negative breast cancer patients. Error bars represent the mean ± SD of three independent experiments. *P < 0.05; **P < 0.01; ***P < 0.001.

By analysis the TCGA published profiles of breast cancer, we found that overexpression of TRIM47 was not only associated with relapse-free survival time (*P* = 0.0031) but also with distant metastasis-free survival time (*P* = 0.0035) compared with lower TRIM47 expression in breast cancer patients (Fig. 1A). Further analysis we found that the mRNA expression of TRIM47 was significantly overexpression in breast cancer compared to normal tissues. Importantly, the mRNA expression of TRIM47 was significantly overexpression in triple-negative breast cancer compared to non-triple-negative breast cancer (Fig. 1B). Interestingly, western blotting and published profiles analyses revealed that TRIM47 was significantly overexpressed in triple-negative breast cancer tissues and in triple-negative breast cancer cell lines at protein levels, compared with the non-triple-negative breast cancer cells and non-triple-negative breast cancer specimens, both in protein and mRNA levels (Fig. 1C–E, Supplemental Fig. 7), suggesting that TRIM47 is upregulated and correlates with poor prognosis in triple-negative breast cancer.

### TRIM47 was significantly negatively correlated with BRCA1 in triple-negative breast cancer

**Fig. 2:**
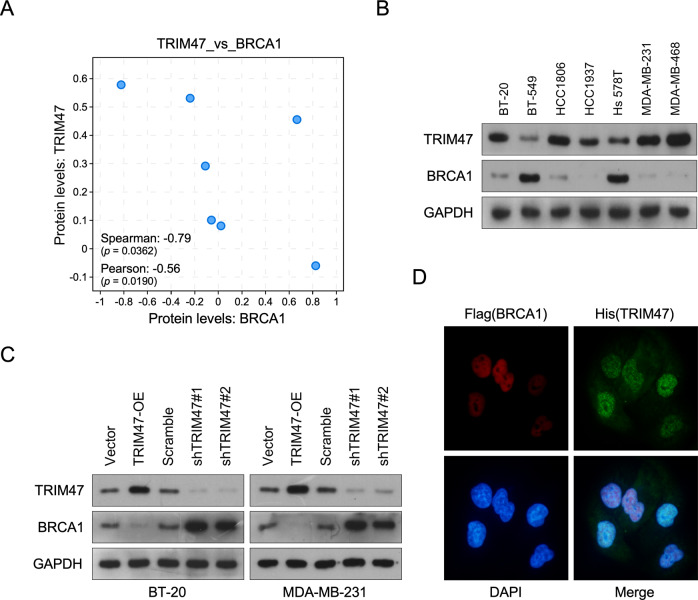
TRIM47 was significantly negatively correlated with BRCA1 in triple-negative breast cancer. **A** Correlation analysis of protein levels of TRIM47 and BRCA1 in public dataset. **B** WB analysis of TRIM47 and BRCA1 expression in the indicated cells. GAPDH served as a loading control. **C** WB analysis of TRIM47 and BRCA1 expression in the indicated cells. GAPDH served as a loading control. **D** Immunofluorescence staining of BRCA1 and TRIM47 in the indicated cells.

It has been reported that breast cancer patients with BRCA1 mutation was mostly found in triple-negative breast cancer. Furthermore, there are similarities in phenotype and molecular level between triple-negative breast cancer and breast cancer with BRCA1 mutation, such as ER negative, CK5/6, EGFR positive, Ki67 high positive, and p53 mutation [18]. Therefore, we analysis the correlation between TRIM47 and BRCA1 in triple-negative breast cancer. Interestingly, the TRIM47 gene is located at the 17q24-25 locus of chromosome 17, while chromosome 17 multibody mutation is frequently reported in invasive breast cancer. By analyzing the copy number of TRIM47 gene in triple-negative breast cancer samples of TCGA database, we found that TRIM47 contains up to 41% gene amplification or mRNA high level in triple-negative breast cancer (Supplemental Fig. 1). Although TRIM65 also has a large proportion of amplification, prognosis analysis shows that there is no significantly correlation between TRIM65 overexpression and prognosis of breast cancer (Supplemental Fig. 2). Published profiles dataset and western blotting analyses both revealed that TRIM47 was significantly reverse correlation between the protein level of TRIM47 and BRCA1 (Fig. 2A, B). Furthermore, we established BT-20 and MDA-MB-231 cells which stably overexpressed TRIM47 and silenced for TRIM47 (Fig. 2C, Supplemental Fig. 7). As expected, overexpression of TRIM47 inhibited but downregulation of TRIM47 induced the protein levels of BRCA1. Immunofluorescence assay found that cells with high TRIM47 expression showed low BRCA1 expression, but cells with low TRIM47 expression showed high BRCA1 expression (Fig. 2D). These above results suggested that TRIM47 was significantly negatively correlated with BRCA1 in triple-negative breast cancer.

### TRIM47 interacted with and induces proteasome-dependent degradation of BRCA1

**Fig. 3:**
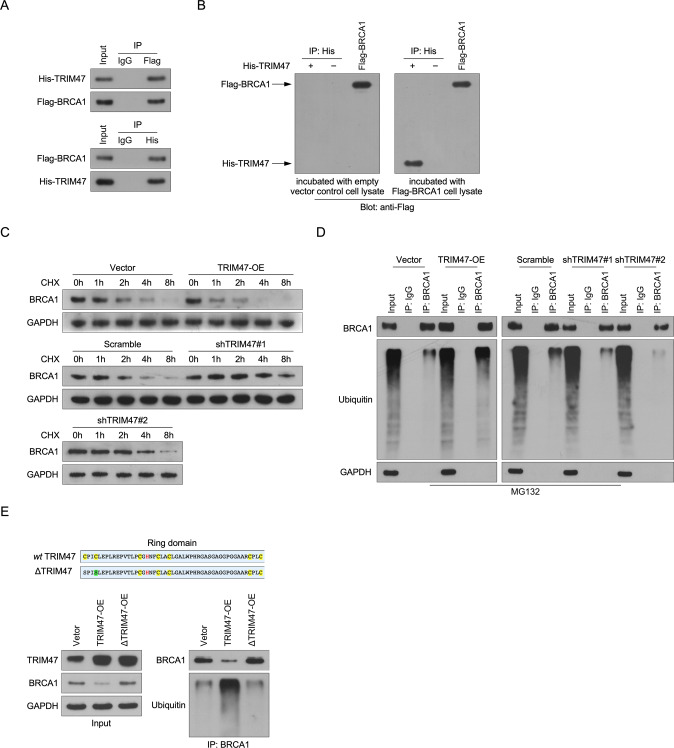
TRIM47 interacted with and induces proteasome-dependent degradation of BRCA1. **A** WB analysis of the half-life of BRCA1 protein in the indicated cells. GAPDH served as a loading control. **B** WB analysis of the polyubiquitin levels of BRCA1 in the indicated cells. **C** WB analysis of the polyubiquitin levels of BRCA1 in the indicated cells. **D** Co-IP assay revealing that endogenous TRIM47 interacted with endogenous BRCA1 in the indicated cells. **E** Immunoprecipitated Flag-tagged BRCA1 was gel-purified, transferred to a membrane, and incubated with His-tagged recombinant TRIM47, then detected using an antibody specific for His or Flag.

Interestingly, by analysis the public data we found that there was no significant correlation between TRIM47 amplification and BRCA1 mutation in triple-negative breast cancer (Supplemental Fig. 3), which suggesting that TRIM47 regulates the decrease of BRCA1 protein expression through other mechanisms. To further investigate the molecular mechanism of TRIM47 in regulating BRCA1, a co-IP assay was performed and showed that TRIM47 forms a complex with BRCA1 in triple-negative breast cancer cells (Fig. 3A). Consistently, we found that TRIM47 interacted with BRCA1 in vivo by performing far-western assay that incubated His-TRIM47 recombinant protein with Flag-BRCA1 cell lysate (Fig. 3B). Furthermore, we found that overexpression of TRIM47 resulted in reduced expression and half-life of BRCA1 but inhibited TRIM47 expression induced expression and half-life of BRCA1 in triple-negative breast cancer cells (Fig. 3C, Supplemental Fig. 8). Previous reports had showed that TRIM47 is a ubiquitinated ligase and mediated SMAD4 ubiquitination modification (Supplemental Figs. 4 and 11), therefore we examined whether TRIM47 regulates BRCA1 protein expression through ubiquitination mechanism. As shown in Fig. 3D, E, ubiquitination experiment shown that overexpression of TRIM47 increased levels of polyubiquitinated BRCA1 however, overexpressing the TRIM47 ring-domain mutant (C9S/C12S) reverse the ubiquitination effect of BRCA1. These results suggested that TRIM47 interacted with and induces proteasome-dependent degradation of BRCA1.

### TRIM47 abolishes the BRCA1-dependent signaling pathway

**Fig. 4:**
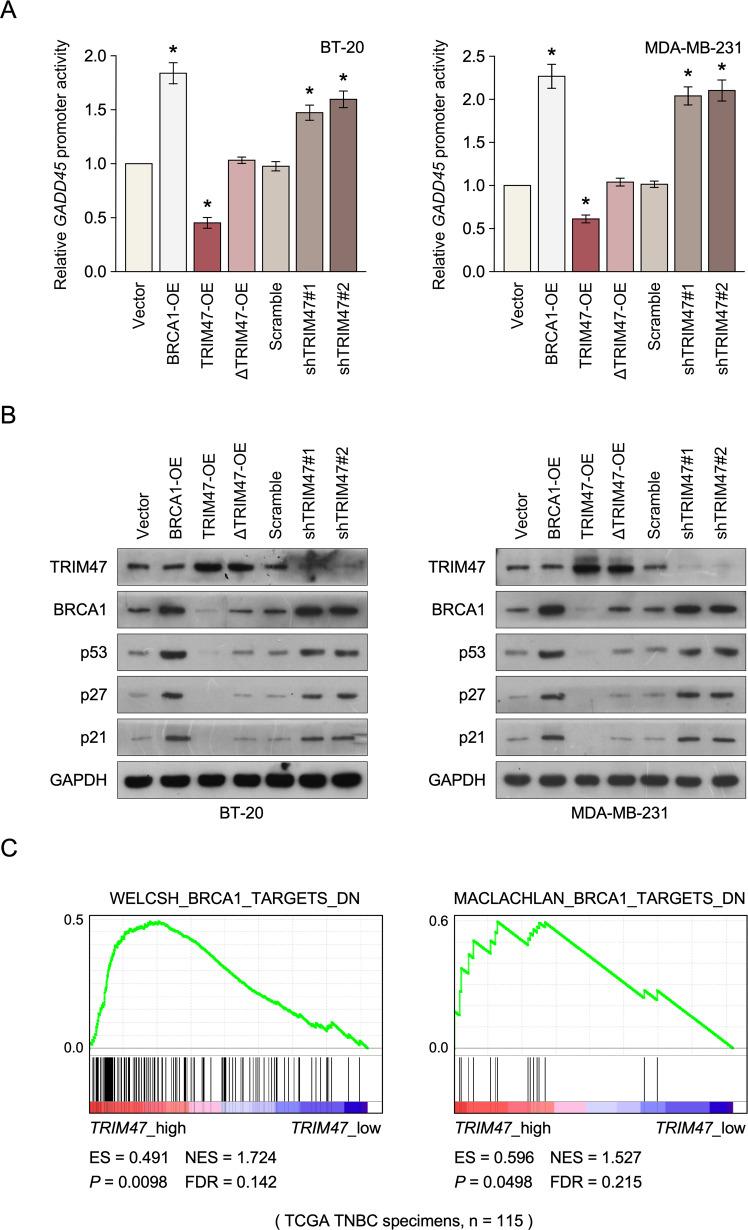
TRIM47 abolishes the BRCA1-dependent signaling pathway. **A** Relative luciferase activity analyses in the indicated cells. **B** WB analysis of TRIM47, BRCA1, P53, P27, P21 protein in the indicated cells. GAPDH served as a loading control. **C** GSEA analysis showing that TRIM47 expression was inversely correlated with BRCA1 target gene signatures in TCGA published TNBC datasets. *P < 0.05.

It has been reported that overexpression of BRCA1 induces GADD45 promoter activation, a p53-regulated and stress-inducible gene [24]. Herein, we examined whether TRIM47 participate in the regulation of GADD45 transcription level. As shown in Fig. 4A, overexpression of BRCA1 in triple-negative breast cancer demonstrate significantly activation levels of the GADD45 promoter, but had no effect on the protein expression level of BRDA1 and TRIM47 (Supplemental Figs. 5 and 11). However, the transcription level of the GADD45 was significantly reversed by TRIM47 overexpression, and increase by inhibition of TRIM47 expression in triple-negative breast cancer cells. Moreover, the downstream gene expression of BRCA1, such as p53, p27, p21 was induced in BRCA1 overexpression cell lines, but was reduced in the overexpression of TRIM47 cell lines and increased in TRIM47-deleted cells, both in protein and mRNA levels (Fig. 4B, Supplemental Figs. 8 and 6). As expected, TRIM47-induced downstream protein expression of BRCA1 was abrogated in TRIM47 Ring-domain mutant overexpressing cells (Fig. 4B). The inverse correlation between TRIM47 expression and BRCA1 pathway was further determined in a gene set enrichment analysis (GSEA) of triple-negative breast cancer specimens (Fig. 4C).

### TRIM47 reverse homologous recombination via inhibit BRCA1 pathway

**Fig. 5:**
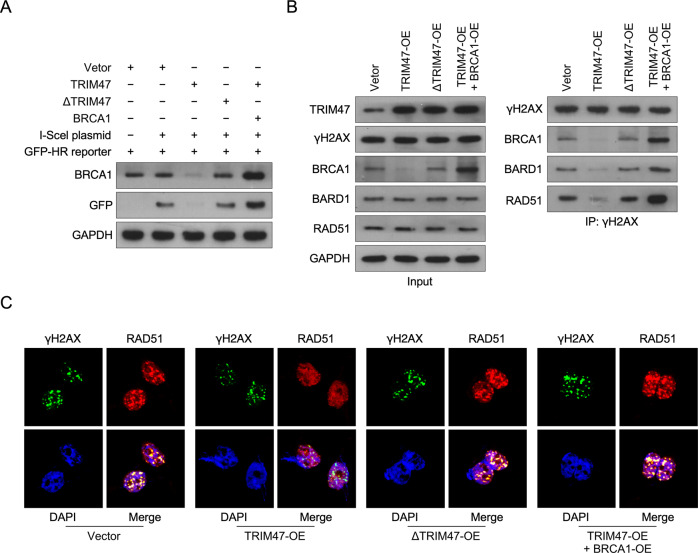
TRIM47 reverse homologous recombination via inhibit BRCA1 pathway. **A** WB analysis of BRCA1, GFP protein in the indicated cells. GAPDH served as a loading control. **B** Co-IP assay revealing that endogenous TRIM47 interacted with endogenous BRCA1, BARD1, RAD51, rH2A in the indicated cells. **C** Immunofluorescence staining of RAD51, rH2A, and TRIM47 in the indicated cells.

Previously study reported that by using the I-SceI system can induce homologous recombination in mammalian chromosomes of saccharomyces cerevisiae [25]. We performed the I-SceI system in order to examine the effect of TRIM47 on BRCA1-induced homologous recombination. Firstly, we found that overexpression of TRIM47 significantly inhibit the recombination events but was abrogated in TRIM47 Ring-domain mutant overexpressing cells (Fig. 5A and Supplemental Fig. 9). Furthermore, overexpression of BRCA1 significantly enhance the recombination effect in TRIM47-transduced cells (Fig. 5A). Reciprocal co-immunoprecipitation (co-IP) analyses demonstrated that overexpression of TRIM47 reduced the interaction of DNA damage repair protein rH2Ax with BRCA1, BRDA1, and RAD51; however, overexpression of BRCA1 dramatically increased the rH2Ax/BRCA1/BRDA1/RAD51 association, but was abrogated in TRIM47 Ring-domain mutant overexpressing cells (Fig. 5B and Supplemental Fig. 9). Importantly, immunofluorescence assay also found that overexpression of TRIM47 reduced nuclear co-localization of rH2Ax and RAD51, however, overexpression of BRCA1 dramatically increased the rH2Ax/RAD51 nuclear association, but was abrogated in TRIM47 Ring-domain mutant overexpressing cells (Fig. 5C). These results suggested that TRIM47 reverse homologous recombination via inhibit BRCA1 pathway.

### BRCA1 inhibits TRIM47 overexpression-induced Olaparib sensitive in triple-negative breast cancer

**Fig. 6:**
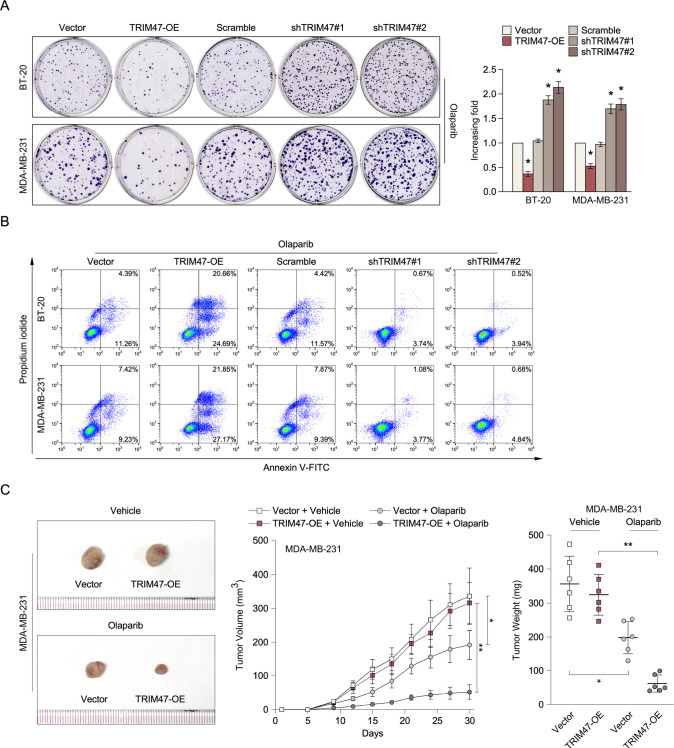
Overexpression of TRIM47 induced Olaparib sensitive in triple-negative breast cancer. **A** Representative micrographs (left) and quantification (right) of crystal violet stained cell colonies formed by the indicated glioma cell lines, 10 days after inoculation. **B** Annexin V-FITC and PI staining of the indicated cells treated with cisplatin for 24 h. Each bar represents the mean ± SD of three independent experiments. **C** Representative tumor-bearing mice (left) and tumor volume (middle) and tumor weights (right) of the subcutaneous tumor model treated as indicated. *P < 0.05; **P < 0.01.

**Fig. 7:**
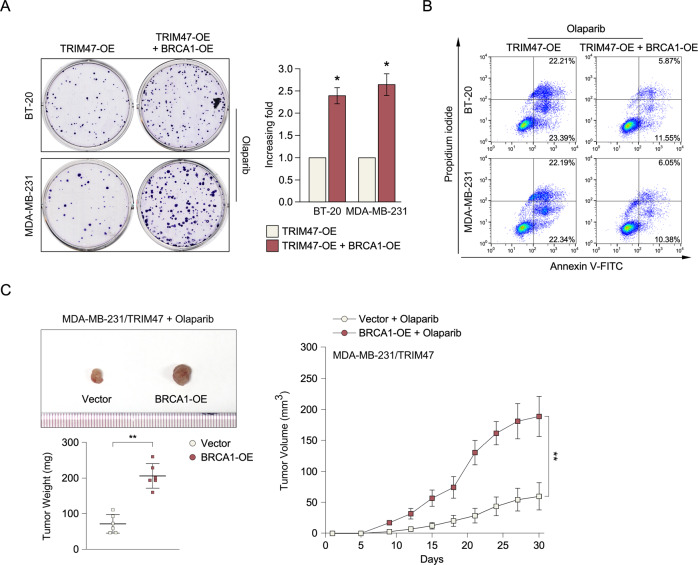
BRCA1 inhibits TRIM47 overexpression-induced Olaparib sensitive in triple-negative breast cancer. **A** Representative micrographs (left) and quantification (right) of crystal violet stained cell colonies formed by the indicated glioma cell lines, 10 days after inoculation. **B** Annexin V-FITC and PI staining of the indicated cells treated with cisplatin for 24 h. Each bar represents the mean ± SD of three independent experiments. **C** Representative tumor-bearing mice (up) and tumor volume (down) and tumor weights (right) of the subcutaneous tumor model treated as indicated. *P < 0.05; **P < 0.01.

It has been reported that Olaparib is an oral poly (ADP-ribose) polymerase inhibitor with activity in germline BRCA1 and BRCA2 (BRCA1/2)-associated breast cancers. To further investigate whether TRIM47 involved in Olaparib sensitive via regulating BRCA1 pathway. As shown in Fig. 6A and B, TRIM47 overexpression conferred Olaparib sensitive in triple-negative breast cancer cell lines, but inhibited TRIM47 conferred Olaparib resistance in triple-negative breast cancer cell lines, as determined by colony formation and Annexin V/PI assay, compared with vector-transformed cells. In agreement with the in vitro results, in vivo subcutaneous tumor model experiments showed that TRIM47 overexpressing cells formed smaller tumors compared with vector-transformed cells in Olaparib treatment group, but there was no significant change in the vehicle group (Fig. 6C). Strikingly, we found that overexpression of BRCA1 significantly reverse the effect of TRIM47 on Olaparib sensitive in triple-negative breast cancer (Fig. 7A–C). Taken together, these results indicate that TRIM47 conferred Olaparib sensitive of triple-negative breast cancer and BRCA1 inhibits TRIM47 overexpression induced Olaparib sensitive both in vitro and in vivo.

### Clinical relevance of TRIM47 and BRCA1 in human triple-negative breast cancer

**Fig. 8:**
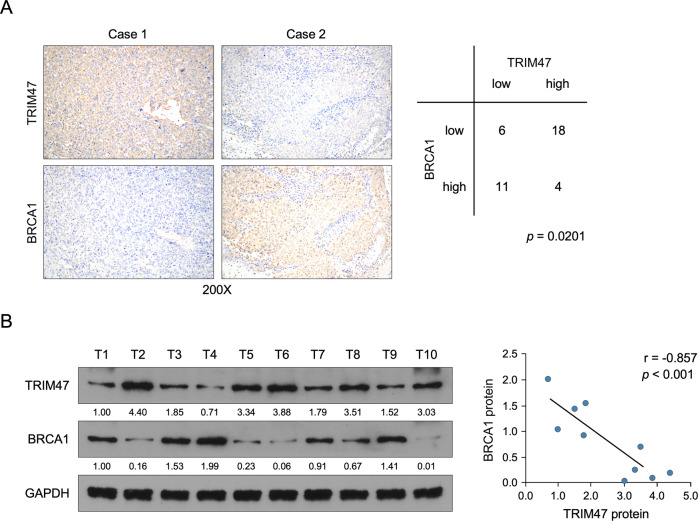
Clinical relevance of TRIM47 and BRCA1 in human triple-negative breast cancer. **A** TRIM47 levels were negatively associated with BRCA1 expression in 39 primary TNBC specimens. *P* = 0.0201. **B** Western blotting of TRIM47 and BRCA1 in 10 cases of freshly TNBC tissues. GAPDH was used as the loading control.

Finally, we examined the correlation of TRIM47 and BRCA1 in collected clinical breast cancer samples. Analysis of 39 human triple-negative breast cancer tissue specimens using IHC analysis showed that TRIM47 expression was inverse correlated with the expression levels of BRCA1 (*P* = 0.021) (Fig. 8A). Consistently, western blot assay showed that TRIM47 was inversely correlated with the expression levels of BRCA1 (*r* = −0.857, *P* < 0.001) in 10 freshly collected clinical triple-negative breast cancer samples (Fig. 8B and Supplemental Fig. 10). Collectively, these results support the notion that overexpression of TRIM47 reduces BRCA1 expression and ultimately leading to Olaparib sensitive in triple-negative breast cancer.

## Discussion

Consistent with TNBC, breast cancer patients who carried BRCA1-deficient is sensitive to DNA damage drugs such as platinum. PARP inhibitors, which have been shown to play a major role in the early clinical trials of BRCA-deficient breast cancer, can enhance the efficacy of TNBC in combination with chemotherapeutic drugs [26]. However, the detection rate of BRCA1/2 mutant germline mutation in TNBC is about 10% although BRCA-deficient cells are highly sensitive to platinum drugs [27]. It suggests that there may be other mechanisms to regulate the downregulation of BRCA1 protein level in TNBC. In the present study, we found that TRIM47 was upregulated TNBC and correlated closely with poorer survival in TNBC. Moreover, TRIM47 directly interacts with BRCA1 and induces ubiquitin ligase-mediated proteasome turnover of BRCA1, subsequently leads to a decrease of BRCA1 protein levels in TNBC. And the downstream gene expression of BRCA1, such as p53, p27, p21 was significantly reduced in the overexpression of TRIM47 cell lines but increased in TRIM47-deleted cells. Taken together, our results demonstrated a novel precise molecular mechanism by which downregulation of BRCA1 protein level in TNBC.

Ubiquitination is one of the important post-translational modifications in eukaryotic cells in order to regulate cell physiological functions [28, 29]. Ubiquitin mediated proteolysis pathway plays an important role in eliminating short-lived regulatory proteins, including proteins related to cell cycle regulation, cell signal transduction, DNA repair, morphogenesis, protein quality control, and transcriptional regulation [30–32]. Therefore, the identification and targeting of E3 ligases involved in the regulation of tumor related-proteins have become the current focus of cancer research. TRIM47, a member of E3 ubiquitin ligase, was identified by serial analysis of gene expression (SAGE) in astrocytoma and codes for a novel ring finger B-box coiled-coil (RBCC) protein [21]. It has been previously reported that TRIM47 is a candidate oncogenic gene in regulating neoplastic processes. For example, increased expression of TRIM47 is a negative prognostic predictor and correlates with poor prognosis in human prostate cancer [22], non-small cell lung carcinoma [23], and gastric cancer. Furthermore, overexpression of TRIM47 accelerates many tumor progressions by regulation of aerobic glycolysis [33], Wnt/β-catenin pathway [34], PI3K/Akt pathway [35], and degradation of SMAD4 [36]. However, the correlation between TRIM47 and olaparib resistance in breast cancer remains unclear. In our current study, we demonstrate that overexpression of TRIM47 was significantly correlated with poor prognosis in triple-negative breast cancer. Moreover, we found that overexpression of TRIM47 in TNBC cells confers an exquisite sensitivity to olaparib, an inhibitor of poly-(ADP-ribose)-polymerase (PARP), but TRIM47 inhibition significantly confers TNBC cells resistance to olaparib both in vitro and in vivo. Taken together, our results suggesting that TRIM47 might be a potential predictive marker for the response to olaparib in TNBC and targeting TRIM47 may be a promising prognostic factor and a valuable therapeutic target for TNBC.

Although previous studies reported that TRIM47 was overexpression in several cancers, but, the mechanism of TRIM47 upregulation in cancer remains unknown. TRIM47 was localized to 17q24-25, a region that is frequently gained or amplified in a number of other tumor types [21]. Interestingly, by analysis the copy Number Variation (CNV) of TRIM47 in the cbioportal dataset (http://www.cbioportal.org/) and we found that there are exhibited a high gene amplification rate of 41% of TRIM47 in breast cancer, suggesting that the overexpression of TRIM47 in breast cancer may be associated with genomic amplification. Furthermore, we found that large amounts of NF-κB and Stat3 were recruited to the promoter region of TRIM47, according to chromatin immunoprecipitation sequencing tracks in the University of California Santa Cruz Genome Browser (http://genome.ucsc.edu/cgi-bin/hgGateway). Therefore, further studies are necessary to determine whether TRIM47 upregulation in breast cancer is attributable to genomic amplification or NF-κB/Stat3-mediated transcriptional upregulation.

In summary, TRIM47 was markedly upregulated in TNBC cells and a positive correlation was evident between TRIM47 expression and metastasis-free survival and the relapse-free survival of breast cancer patients. Overexpression of TRIM47 in TNBC cells confers an exquisite sensitivity to olaparib, an inhibitor of poly-(ADP-ribose)-polymerase (PARP), but TRIM47 inhibition significantly confers TNBC cells resistance to olaparib, by directly interacts with BRCA1 and induces ubiquitin ligase-mediated proteasome turnover of BRCA1, subsequently leads to a decrease of BRCA1 protein levels in TNBC. Elucidation of the biologic function and regulation mechanism of TRIM47 in BRCA1-defection TNBC progression will advance our knowledge of the mechanisms underlying TNBC chemo-resistance and establish TRIM47 as a potential therapeutic target for overcoming drug resistance in patients with TNBC.

## Materials and methods

### Cell culture

The human breast cancer cell lines Hs578Bst, MCF7, MDA-MB-453, T-47D, ZR-75-1, ZR-75-30, BT-20, BT-549, CAMA-1, HCC1806, HCC1937, Hs578T, MDA-MB-231, and MDA-MB-468 were cultured according to the manufacturer’s instruction. Short tandem repeat (STR) profiling were used and authenticated in all cell lines.

### Chemical reagents

Olaparib was purchased from https://www.selleck.cn/ (AZD2281).

### Tissue specimens and immunohistochemistry

This study was conducted on a total of 39 paraffin-embedded TNBC samples. 39 cases of paraffin-embedded TNBC was used to detect the expression of TRIM47 using Anti-TRIM47 antibody (ab72234) and Anti-BRCA1 antibody [MS110] (ab16780). For the use of these clinical materials for research purposes, prior patient consent and approval from the Institutional Research Ethics Committee were obtained. The 39 specimens were histopathologically and clinically diagnosed in Integrated Hospital of Traditional Chinese Medicine, Southern Medical University. 10 freshly TNBC tissues were histopathologically and clinically diagnosed in the First Affiliated Hospital of Sun Yat-sen University were frozen and stored in liquid nitrogen until further use.

### Vectors, retroviral infection, and transfection

pMSCV/TRIM47 recombinant plasmid was generated by subcloning the PCR-amplified human TRIM47 coding sequence into pMSCV vector. pMSCV/BRCA1 recombinant plasmid was generated by subcloning the PCR-amplified human BRCA1 coding sequence into pMSCV vector. TRIM47 ring-domain mutant (C9S/C12S) plasmid was generated by using QuikChange ® Site-Directed Mutagenesis Kit according to manufacturer’s instructions. To silence endogenous TRIM47, two siRNA oligonucleotides were cloned to generate pSuper-retro-shTRIM47#1, shTRIM47#2, respectively. The primers using in this study were listed in Supplementary Table 1. Transfection of siRNA or plasmids was performed using the Lipofectamine 3000 reagent (Invitrogen, Carlsbad, CA) according to the manufacturer’s instruction. Stable cell lines expressing TRIM47 or shTRIM47#1, shTRIM47#2 were selected for 10 days with 0.5 μg/ml puromycin 48 h after infection.

### Immunoprecipitation analysis

Immunoprecipitation assay was performed according to described previously [37]. Lysates were incubated with Flag or His affinity beads (Sigma-Aldrich). The agarose beads were washed with wash buffer. Then the elutions were detected using appropriate antibodies.

### Far western blotting

The indicated proteins were immunoprecipitated by Flag-tag affinity gel (Sigma-Aldrich, St. Louis, MO) and resolved by SDS-PAGE, and the proteins were transferred to PVDF membrane. The membrane was then blocked in 10% skimmed milk for 1 h at 4 °C. As indicated, recombinant His-TRIM47 or Flag-BRCA1 was added at 1 μg/ml and incubated at 4 °C for 18 h. The blot was then washed 6 times with TBST and subjected to WB analysis using anti-His or anti-Flag antibody (Sigma-Aldrich, St. Louis, MO).

### Western blotting analysis

Western blotting was performed using antibodies against Anti-TRIM47 antibody (ab72234), Anti-p53 antibody [PAb 240] (ab26), Anti-p21 antibody [EPR362] (ab109520), Anti-p27 KIP 1 antibody [Y236] (ab32034), Anti-rH2AX antibody [SIGMA] (H5912), Anti-BARD1 antibody [2059C4a] (ab50984), Anti-Rad51 antibody [EPR4030(3)] (ab133534), Anti-GFP antibody (ab290), and Anti-BRCA1 antibody [MS110] (ab16780). The membranes were re-probed with an Anti-GAPDH antibody [6C5] - Loading Control (ab8245) as the loading control.

### Quantitative real-time reverse transcription PCR (qPCR)

Total RNA was isolated with TRIzol reagent (Invitrogen) according to manufacturer’s instructions. RNA was reverse-transcribed into cDNA and carried out via Real-time PCR with SYBR Green Master (Roche). The data were assessed based on the threshold cycle (Ct), and calculated as ${2}^{-[{({C_i}\,{\mathrm{of}}\,{\mathrm{gene}})}\,-\,{({C_i}\,{\mathrm{of}}\,{\mathrm{GAPDH}})}]}$, which was normalized to GAPDH expression. All primers are listed as Supplementary Table S1.

### Immunofluorescence (IF) staining

IF staining was carried out on indicated cells grown on coverslips using Anti-Rad51 antibody [EPR4030(3)] (ab133534), Anti-rH2AX antibody [SIGMA] (H5912), and FITC-conjugated goat anti-mouse secondary antibody (Jackson Immuno Research). The images were captured using the AxioVision Rel.4.6 computerized image analysis system (Carl Zeiss).

### Colony formation assay

Colony formation assay was performed according to described previously [38]. Indicated cells were plated on 60 mm plates (0.5 × 103 cells per plate) and incubated at a level of 5% CO_2_ at a temperature of 37 °C for 2 weeks. The colonies were stained with 1.0% crystal violet for 30 s after fixation with 10% formaldehyde for 5 min. Then the cells were counted and analyzed.

### Luciferase activity assay

Luciferase activity assay was performed according to described previously [38]. Luciferase reporter plasmid and pRL-TK Renilla plasmid were transfected into indicated cells. After 48 h, the cells were lysis and measured using a Dual Luciferase Reporter Assay (Promega) according to the manufacturer’s instructions.

### Annexin-V assay

Annexin-V assay was performed according to described previously [39]. For evaluation of apoptosis, PE Annexin V Apoptosis Detection Kit I (BD Pharmingen) was used. Briefly, 1 × 10^6^ indicated treated cells were collected and washed with PBS and the Annexin-V binding solution, subsequently added 150 μl of an Annexin-V antibody in Binding Buffer and incubated for 15 min, followed by addition of 1.5 μl of PI at 1 mg/ml and a further incubation for 5 min at room temperature in the dark. Cell morphology was assessed by phase-contrast microscopy. Percentage of apoptosis was analyzed with an EPICS XL flow cytometer (Beckman-Coulter). Each sample was analyzed in triplicate.

### Tumor xenografts

Six-week-old BALB/c-nu mice were purchased from the Center of Experimental Animal of Guangdong Province (Guangdong, China), and approval for the experiments was obtained from the Institutional Animal Care and Use Committee.

To form tumor xenografts in female six-week-old BALB/c nude mice, Vector or TRIM47-transfected-MDA-MB-231 cells (1 × 10^7^) were injected subcutaneously in the back under aseptic conditions with or without Olaparib. Tumor size was measured using callipers every week, and tumor volume was calculated according to the following equation: (long axis × short axis2)/2. The xenograft tumors were removed on the sixth week after the injection and weighed after dissection.

### Data processing and visualization

For the relationship between TRIM47 and the OS, RFS, and MFS of breast cancers, Kaplan–Meier Plotter ((http://kmplot.com/analysis) was used. The datasets are available in The Cancer Genome Atlas (TCGA) (https://tcga-data.nci.nih.gov/tcga/). Gene set enrichment analysis (GSEA) was performed on GSEA 2.0.9 (http://www.broadinstitute.org/gsea/).

### Statistics

SPSS 21.0 statistical software (IBM, Armonk, NY, USA) was used to analyze the data. Differences between two variables were evaluated using Student’s t-test. The relationship between TRIM47 expression and the clinicopathological characteristics were carried out using the chi-squared test, log-rank analysis, the Kaplan–Meier method, and the Cox regression model. *P* < 0.05 was considered statistically significant.

## Supplementary information

supplemental figure 1

supplemental figure 2

supplemental figure 3

supplemental figure 4

supplemental figure 5

supplemental figure 6

supplemental figure 7

supplemental figure 8

supplemental figure 9

supplemental figure 10

supplemental figure 11

Supplementary Informations

## Data Availability

The datasets used and/or analyzed during the current study are available from the corresponding author upon reasonable request.
