# Supplementary figures and images for: Overexpression of tripartite motif-containing 47 (TRIM47) confers sensitivity to PARP inhibition via ubiquitylation of BRCA1 in triple negative breast cancer cells
Source: Oncogenesis. 2023 Mar 11;12(1):13. doi: 10.1038/s41389-023-00453-7 (PMC10008536; doi:10.1038/s41389-023-00453-7)

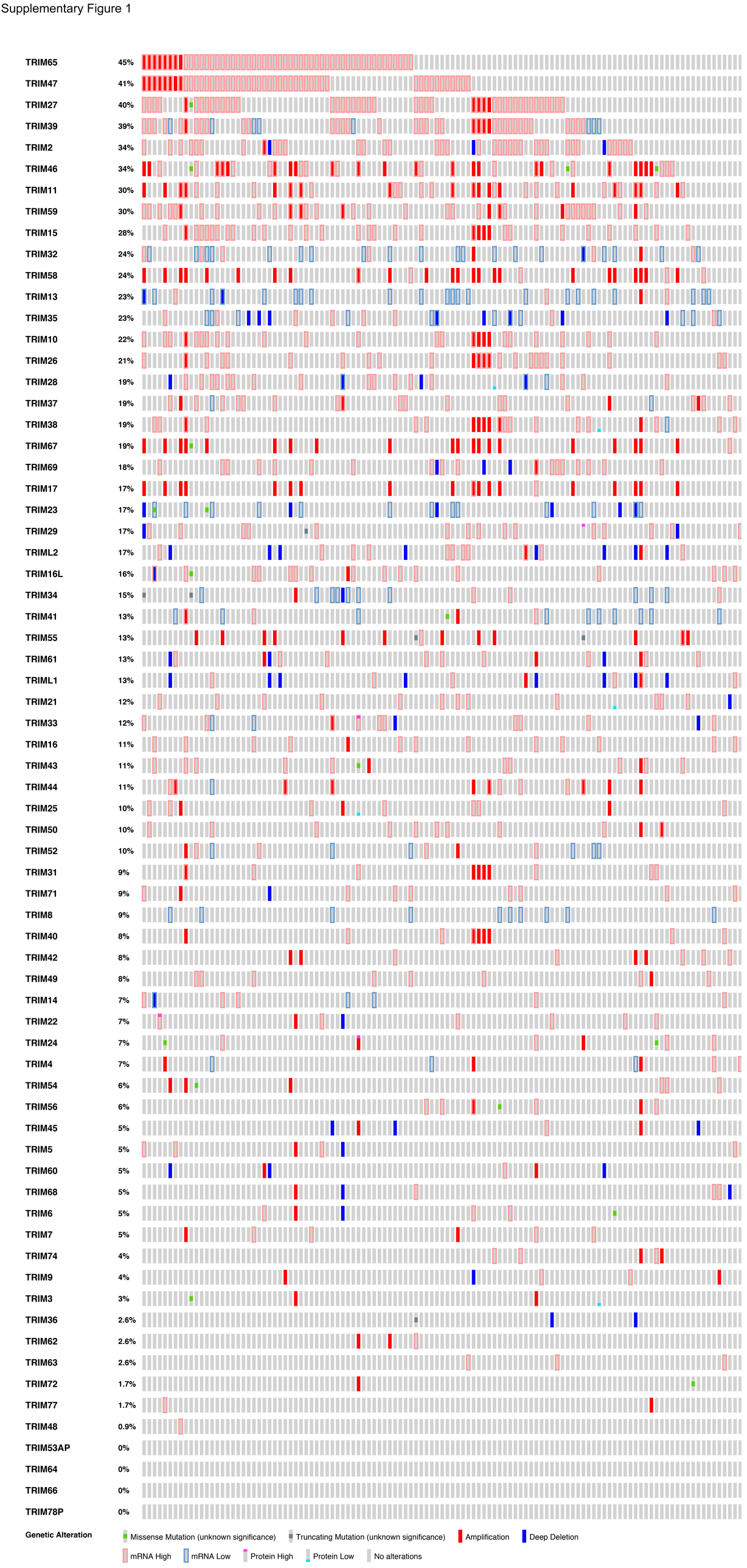

Supplement: Supplementary file 1 — supplemental figure 1 [file 41389_2023_453_MOESM1_ESM.tif]

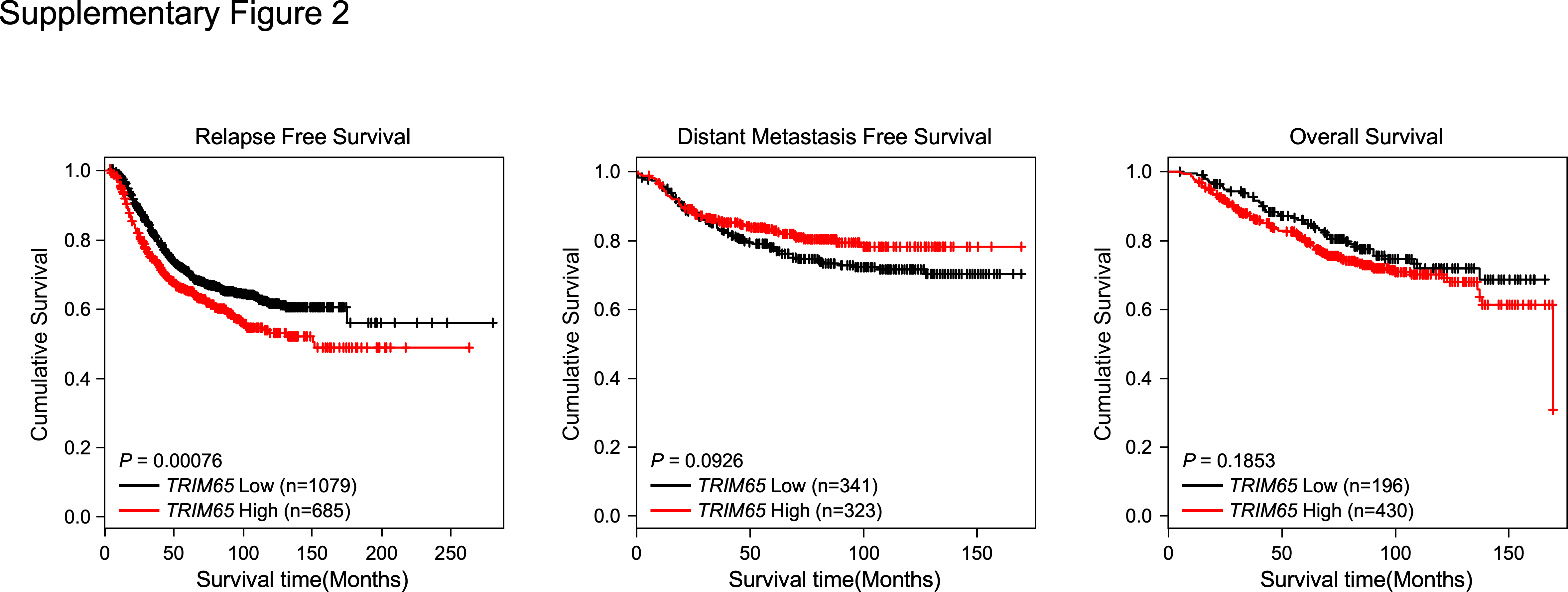

Supplement: Supplementary file 2 — supplemental figure 2 [file 41389_2023_453_MOESM2_ESM.tif]

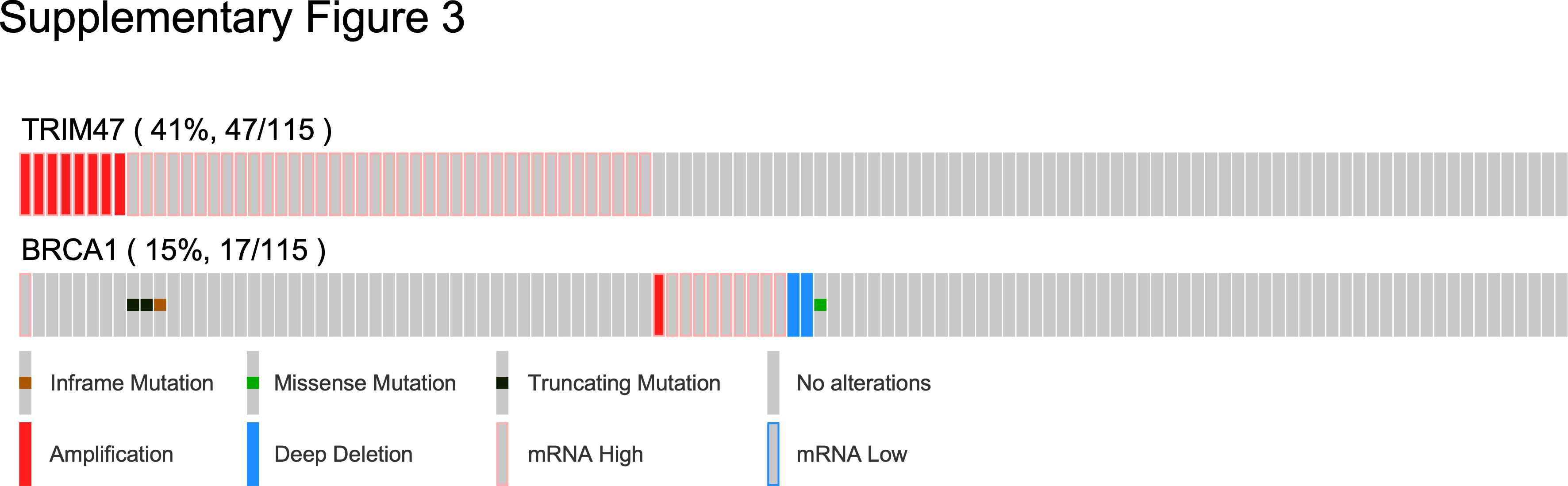

Supplement: Supplementary file 3 — supplemental figure 3 [file 41389_2023_453_MOESM3_ESM.tif]

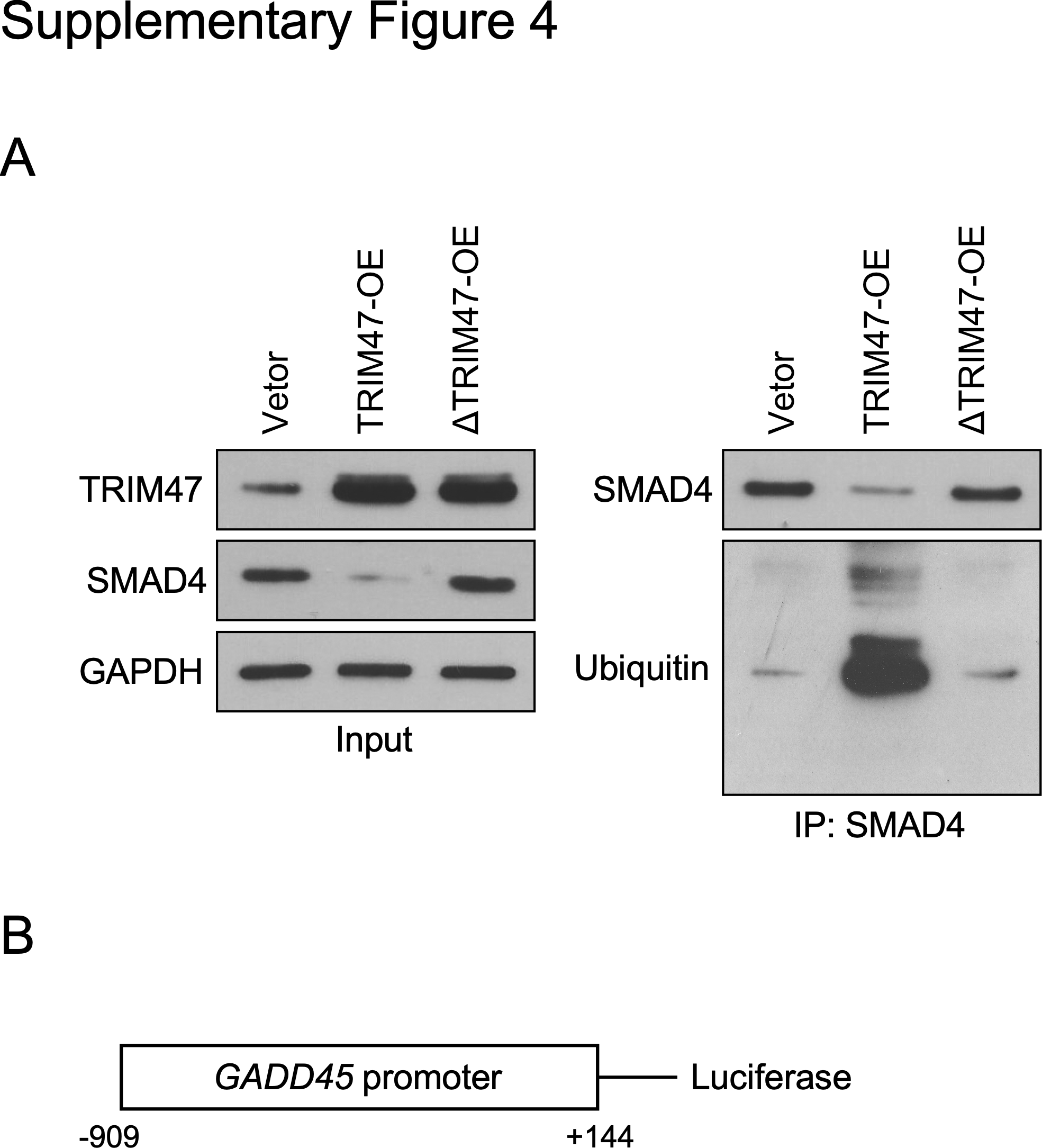

Supplement: Supplementary file 4 — supplemental figure 4 [file 41389_2023_453_MOESM4_ESM.tif]

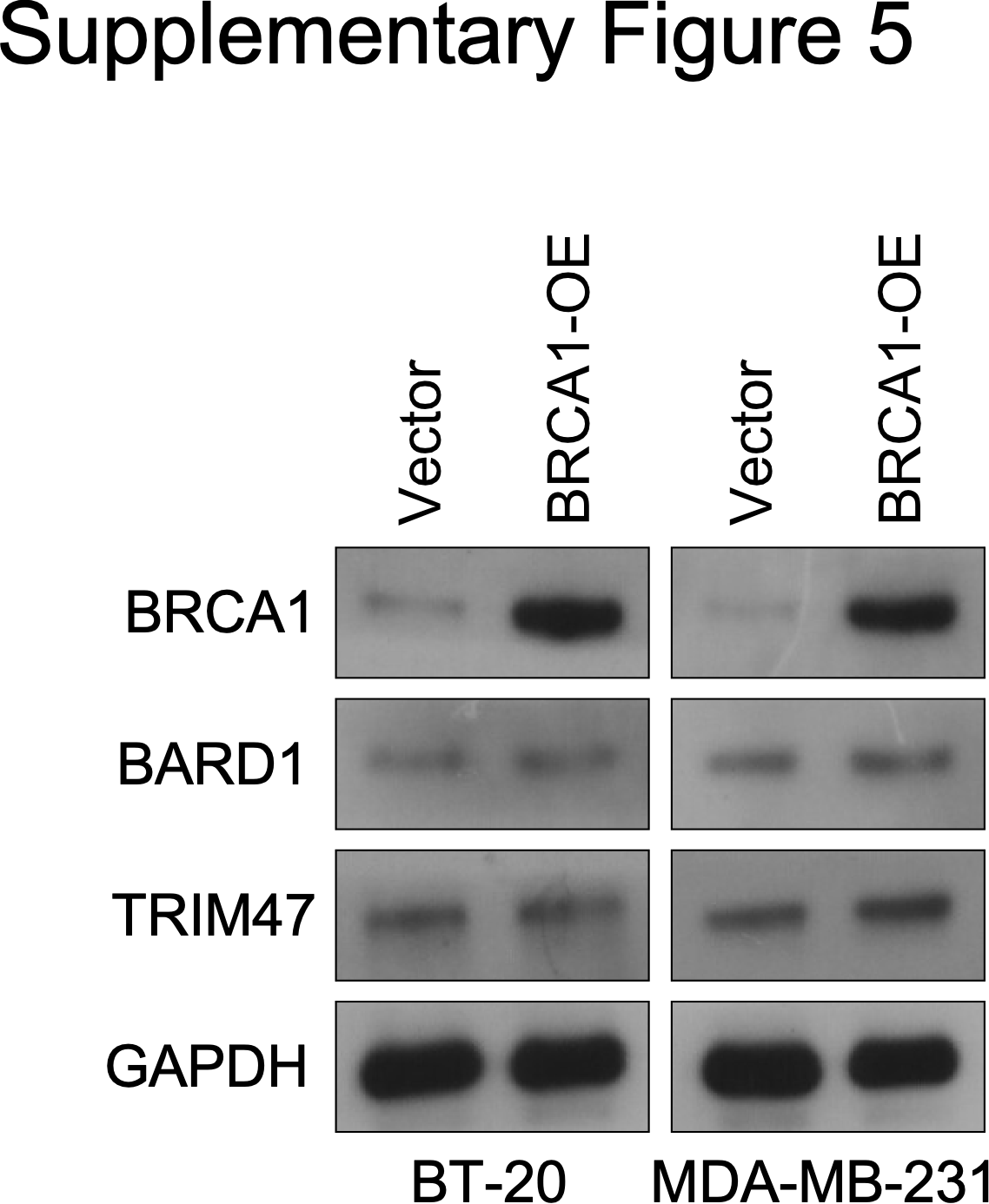

Supplement: Supplementary file 5 — supplemental figure 5 [file 41389_2023_453_MOESM5_ESM.tif]

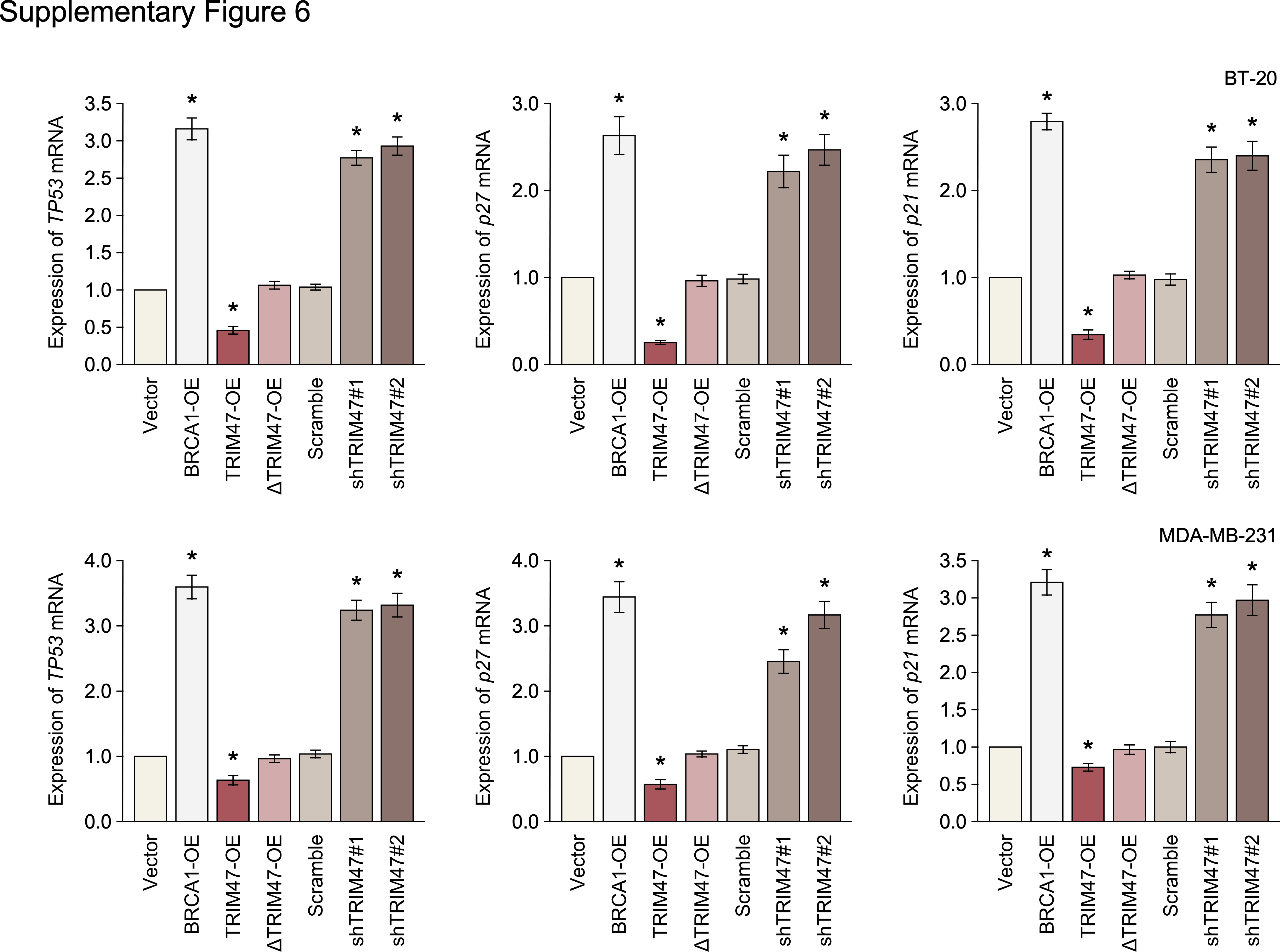

Supplement: Supplementary file 6 — supplemental figure 6 [file 41389_2023_453_MOESM6_ESM.tif]

Supplementary Figure 8

Figure 3c

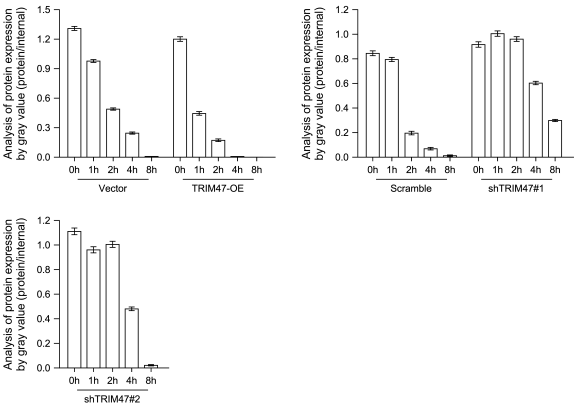

Figure 3e

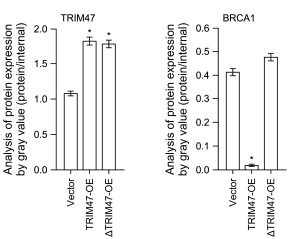

Figure 4b

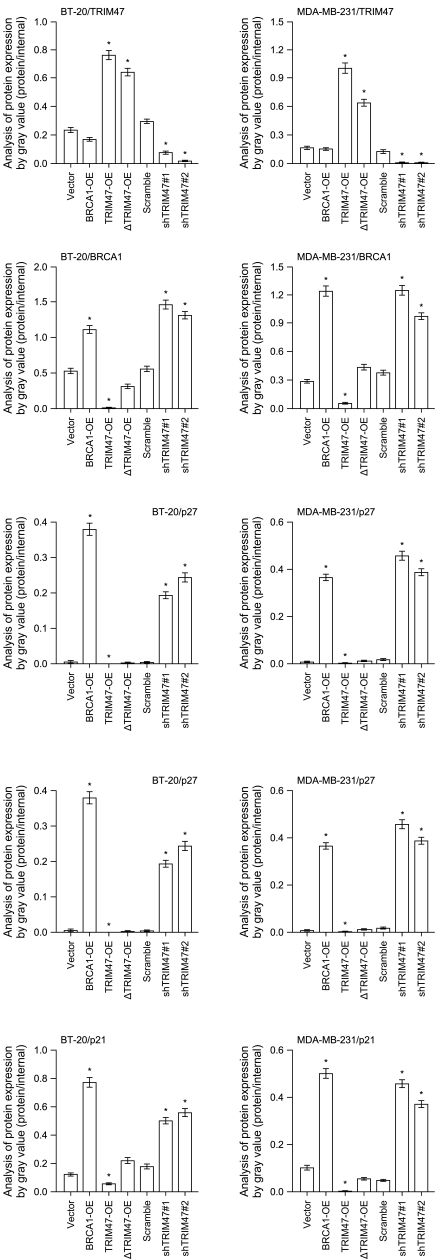

Supplement: Supplementary file 8 — supplemental figure 8 [file 41389_2023_453_MOESM8_ESM.pdf]

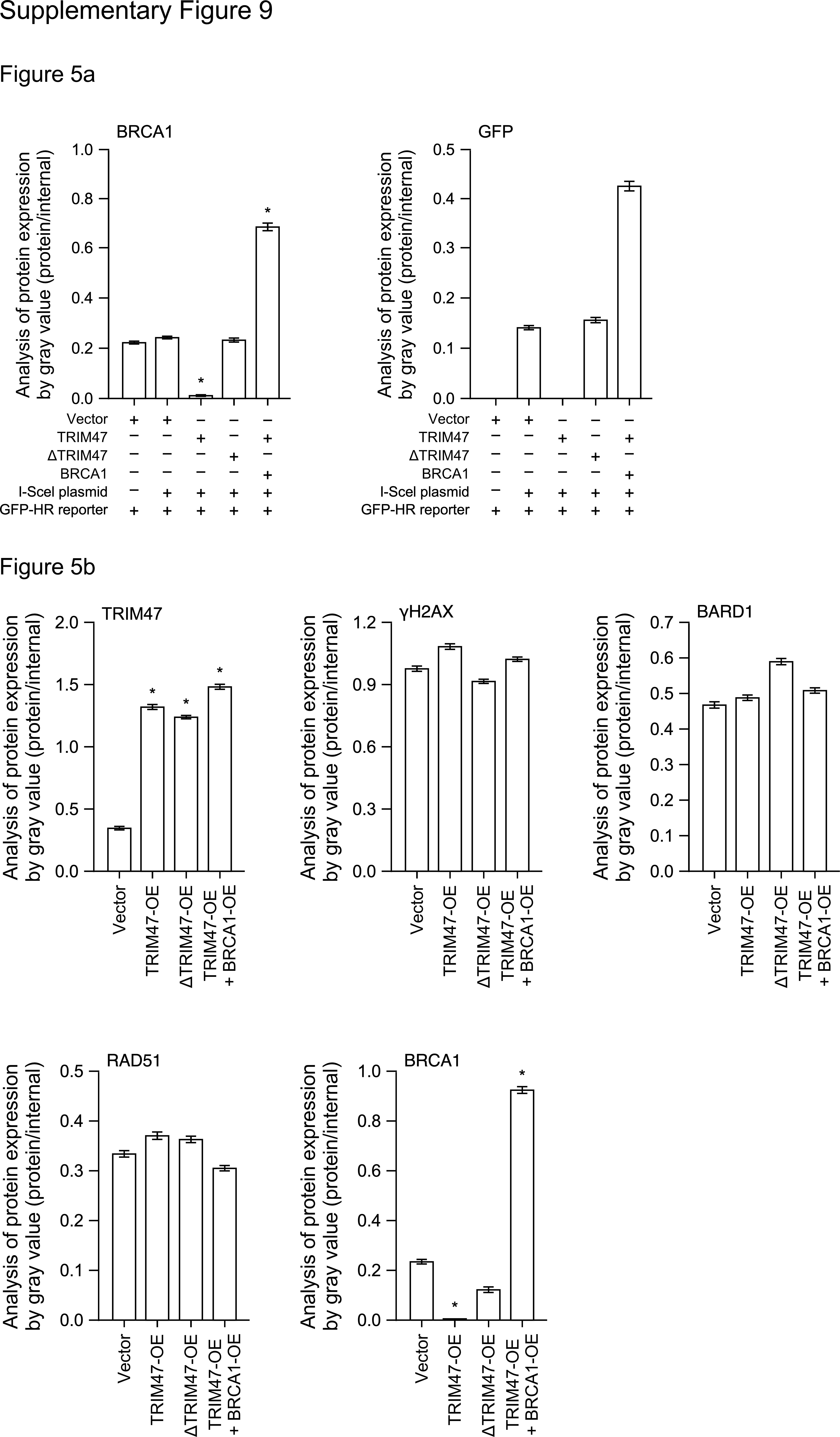

Supplement: Supplementary file 9 — supplemental figure 9 [file 41389_2023_453_MOESM9_ESM.tif]

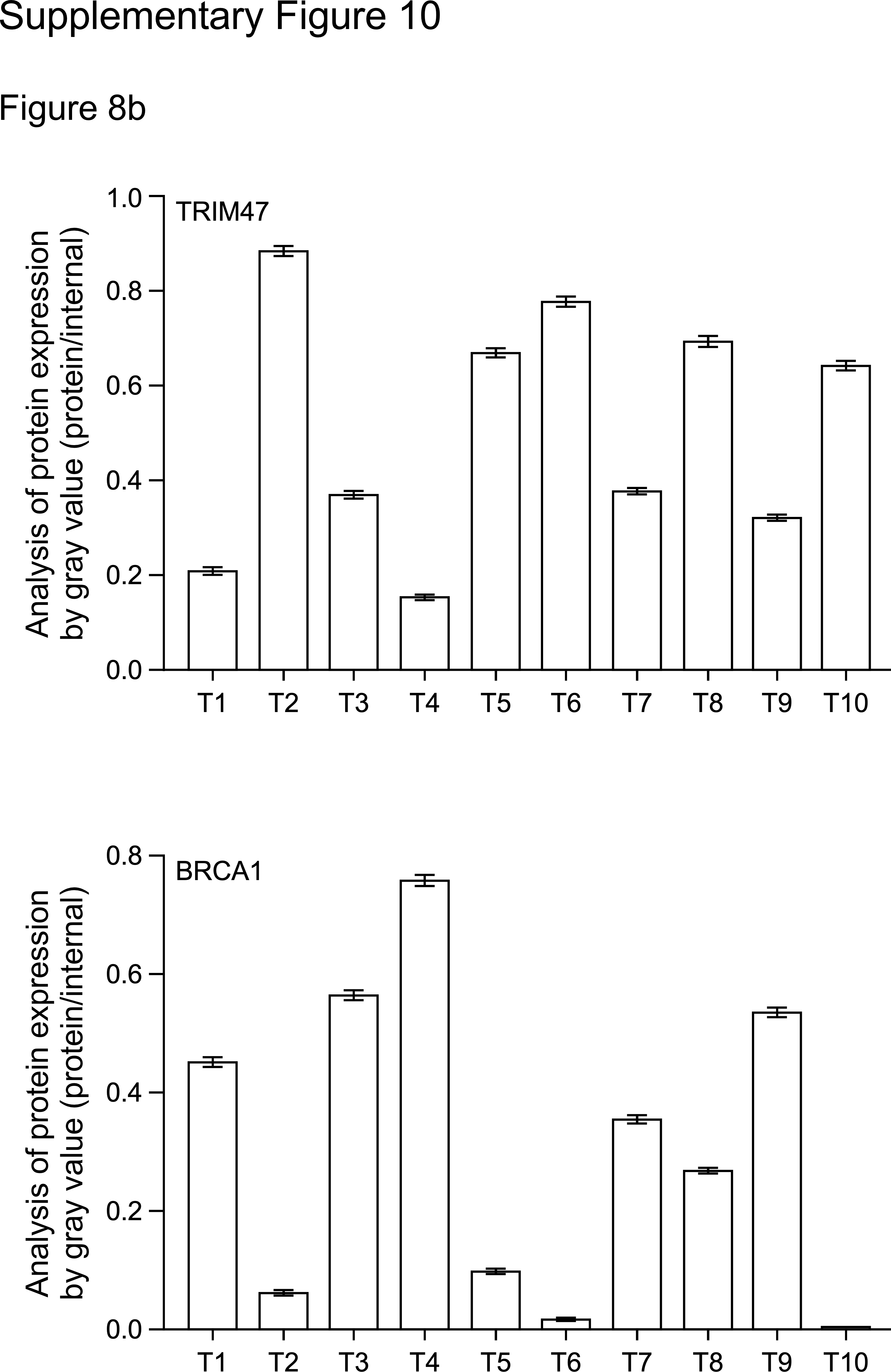

Supplement: Supplementary file 10 — supplemental figure 10 [file 41389_2023_453_MOESM10_ESM.tif]

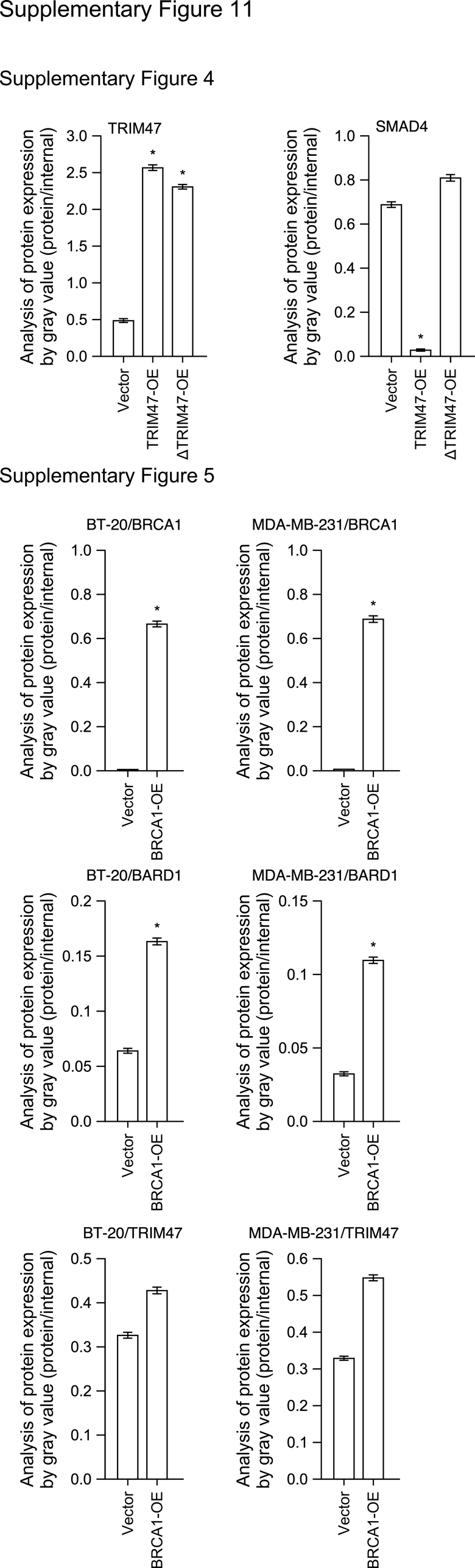

Supplement: Supplementary file 11 — supplemental figure 11 [file 41389_2023_453_MOESM11_ESM.tif]
