# Supplementary material for: Overexpression of tripartite motif-containing 47 (TRIM47) confers sensitivity to PARP inhibition via ubiquitylation of BRCA1 in triple negative breast cancer cells
Source: Oncogenesis. 2023 Mar 11;12(1):13. doi: 10.1038/s41389-023-00453-7 (PMC10008536; doi:10.1038/s41389-023-00453-7)
Supplement: Supplementary file 12 — Supplementary Informations [file 41389_2023_453_MOESM12_ESM.docx]

**Supplemental information**

**Supplementary Figure legends**

**Supplementary Figure** **1**. Analysis the copy Number Variation(CNV) of TRIM47 in the cbioportal dataset.

**Supplementary Figure** **2**. Kaplan-Meier analysis of Relapse-Free Survival and Distant Metastasis Free survival from public dataset for breast cancer patients with low TRIM65 expression or high TRIM65 expression.

**Supplementary Figure** **3**. Analysis the copy Number Variation(CNV) of TRIM47 and BRCA1 in the cbioportal dataset.

**Supplementary Figure** **4**. WB analysis of the polyubiquitin levels of SMAD4 in the indicated cells.

**Supplementary Figure** **5**. WB analysis of BRCA1, BRAD1 and TRIM47 protein in the indicated cells. GAPDH served as a loading control.

**Supplementary Figure** **6**. mRNA analysis of P53, P27, P21 in the indicated cells.

**Supplementary Figure 7-11**.Densitometric analyses of immunoblots .

**Supplemental Tables 1: Primers and Oligonucleotides**

| **Used for subcloning and plasmid construction:** | |
| --- | --- |
| TRIM47 shRNA#1 | GGGAGCTCAGCTTCACCAAAT |
| TRIM47 shRNA#2 | GCAGCTGTTTGGAACCAAAGG |
| **Used for qPCR** |  |
| TRIM47 real time primer up | CTGAGCAGTCCAAAGTCCTGA |
| TRIM47 real time primer dn | CTACGGCTGCACTCTTGATG |
| TP53 real time primer up | CAGCACATGACGGAGGTTGT |
| TP53 real time primer dn | TCATCCAAATACTCCACACGC |
| P21 real time primer up | TGTCCGTCAGAACCCATGC |
| P21 real time primer dn | AAAGTCGAAGTTCCATCGCTC |
| P27 real time primer up | AGGAGGAGATAGAAGCGCAGA |
| P27 real time primer dn | GTGCGGACTTGGTACAGGT |
| GAPDH real time primer up | GGAGCGAGATCCCTCCAAAAT |
| GAPDH real time primer dn | GGCTGTTGTCATACTTCTCATGG |
